# The Developmental Biology of Parasitic Nematodes

**DOI:** 10.1371/journal.ppat.1005328

**Published:** 2016-03-10

**Authors:** James B. Lok

**Affiliations:** Department of Pathobiology, School of Veterinary Medicine, University of Pennsylvania, Philadelphia, Pennsylvania, United States of America; University of Notre Dame, UNITED STATES

My research focuses on understanding the factors that regulate the development of parasitic nematodes (roundworms) during the infective process, in which their larvae pass from the external environment into mammalian hosts, including humans. This research matters because these factors are essential to the survival of a group of pathogenic organisms that collectively cause debilitation, illness, and some deaths in over 1.5 billion people around the globe. In this enormous population—comprising almost one in five people on the planet—parasitic nematodes such as hookworms, roundworms, whipworms, and threadworms complicate pregnancy, impair both physical and cognitive development in children, and sap the productive energies of infected adults. Other parasitic nematodes, such as the Guinea worm and the filariae, cause pain and disfigurement of limbs or excruciating skin disease and potentially blinding lesions of the eye. This problem is underappreciated by both policy makers and the general public in relatively prosperous countries because the diseases involved occur under conditions of extreme poverty—their parasitic agents thrive in communities of people who are food-insecure and who live in substandard housing and with poor sanitation. Parasitic nematodes also contribute to food insecurity by causing anemia, gastroenteritis, and malnutrition in livestock and attendant decrements in milk and meat production.

My primary research subject is the intestinal threadworm, *Strongyloides stercoralis*. Like many other parasitic nematodes, *S*. *stercoralis* develops in contaminated soil, through a series of noninfectious larval stages, into an infectious form, the third-stage larva, which penetrates the skin of its human or canine host and undertakes a complex migration to the small intestine. There, it can cause enteritis and diarrhea in uncomplicated infection and can cause overwhelming and potentially fatal fulminant infection in hosts with compromised immune systems. *S*. *stercoralis* is an important pathogen in its own right, but we study it because some peculiarities of its life cycle allow us to subject the worms to genetic study that would be impossible or impractical in other parasitic nematodes. One such genetic method that my research group has developed for *S*. *stercoralis* is transgenesis—the ability to transfer new genes, sometimes with strategic alterations in their functions, into the organism. Thus far, *Strongyloides* and some related worms are the only parasitic nematodes in which transgenesis has proven feasible. We were able to accomplish this important technical advancement because similarities in the body forms of *Strongyloides* and the free-living nematode *Caenorhabditis elegans* (which is widely used as a model for basic research) allowed us to adapt well-developed techniques for transgenesis in that worm to our parasites.

The basic research questions we address with this method revolve around how *Strongyloides* uses molecular signaling mechanisms within its cells to control the formation of its infective larvae, the behaviors these larvae use to find and invade their hosts, and the processes by which they resume their development to adult parasites within the intestines of these hosts. We took our cues about which of the many signaling mechanisms operating in *Strongyloides* might be important in this infectious process from the wealth of knowledge about how such mechanisms control development of the free-living nematode, *C*. *elegans*. We and our colleagues recently identified a signaling pathway involving steroid hormones in *Strongyloides* that may point the way towards development of new drugs to combat infection by this parasite in humans—a crucial need, given the relatively small number of currently available drugs, and another reason why this research matters. Thus, my research draws heavily upon basic science developed with the *C*. *elegans* model, both for its conceptual underpinnings and for its methodology. This aspect of the research matters because it places the work within the profoundly beneficial movement towards translational biomedical science that has energized and accelerated the flow of vital information from the laboratory bench to the patient bedside, of late.

Finally, the research matters to me personally because, after almost 35 years at it, I am still fascinated by the parasitic worms that I study. A large part of this is the thrill of glimpsing the unseen, of “seeing” the inner workings of organisms that spend some or all of their time living inside of other animals. I also find the worms to be beautiful in their own way. I am never happier than when I can take a precious hour or two away from the cares of administrative duties and teaching commitments to sit alone at the microscope and study them in detail in order to document changes resulting from our genetic manipulations that will constitute the next new finding. Far from a personal conceit, fascination of this type is a driving force behind the best basic science, and so, it matters very much in the fight to eradicate the scourge of parasitic disease from the globe.

**Image 1:**
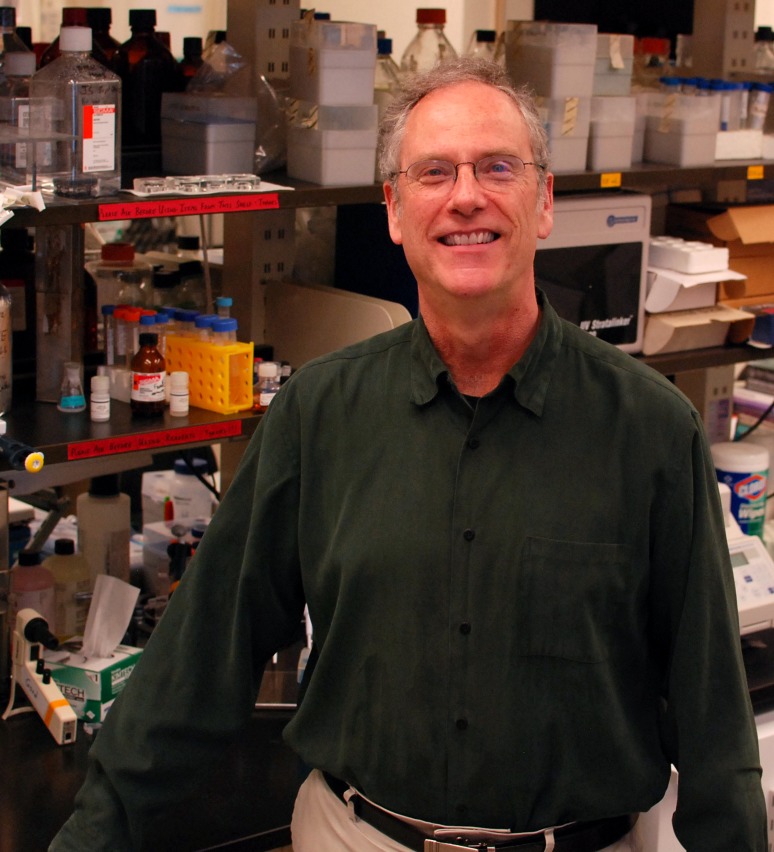
James B. Lok.

